# Antioxidant, Polyphenol, Physical, and Sensory Changes in Myofibrillar Protein Gels Supplemented with Polyphenol-Rich Plant-Based Additives

**DOI:** 10.3390/nu17071232

**Published:** 2025-04-01

**Authors:** Katarzyna Leicht, Charles Odilichukwu R. Okpala, Paulina Nowicka, José Angel Pérez-Alvarez, Małgorzata Korzeniowska

**Affiliations:** 1 Department of Functional Food Products Development, Faculty of Biotechnology and Food Science, Wroclaw University of Environmental and Life Sciences, 37 Chelmonskiego, 50-630 Wroclaw, Poland; katarzyna.leicht@upwr.edu.pl; 2 UGA Cooperative Extension, College of Agricultural and Environmental Sciences, University of Georgia, Athens, GA 30602, USA; charlesokpala@gmail.com; 3 Department of Fruit, Vegetable and Plant Nutraceutical Technology, Faculty of Biotechnology and Food Science, Wroclaw University of Environmental and Life Sciences, 37 Chelmonskiego, 50-630 Wroclaw, Poland; paulina.nowicka@upwr.edu.pl; 4 Agro Food Technology Department, Escuela Politécnica Superior de Orihuela, Miguel Hernández University, Crta. Beniel km. 3.2, E-03312 Orihuela, Spain; ja.perez@umh.es

**Keywords:** functional foods, myofibrillar protein gel, plant-based additives, polyphenol, health-promoting products

## Abstract

Background: Plant-based additives such as blackcurrant juice and pomace, as well as herbal extracts from *Melissa officinalis* and *Centella asiatica*, possess well-established health-promoting properties. This study aimed to investigate how the incorporation of polyphenol-rich plant-based additives into a myofibrillar protein matrix could enhance the nutritional value, antioxidant potential, and sensory quality of novel food gels. Methods: Myofibrillar protein gels were enriched with selected plant-based additives. Antioxidant properties were assessed using the ABTS radical cation decolorization assay, DPPH radical scavenging assay, and the Ferric Reducing Antioxidant Power (FRAP) assay. Polyphenol profiles were determined with emphasis on flavonols, flavan-3-ols, and chlorogenic acids. Physicochemical properties including pH, color, texture, energetic value, dry matter, and ash contents were measured. Sensory evaluation was conducted using consumer preference tests and descriptive sensory profiling. Results: Enriched gels contained bioactive compounds such as catechins, procyanidins, chlorogenic acids, and anthocyanins, whose presence correlated with distinct antioxidant activities. Blackcurrant pomace significantly elevated both total polyphenol content and antioxidant capacity, imparting a vivid red-purple color that influenced consumer perception. *Melissa officinalis* extract enhanced antioxidant potential and introduced a mild, pleasant aroma. *Centella asiatica* extract further improved the nutritional profile and oxidative stability of the gels, demonstrating additive and synergistic effects in both functional and sensory dimensions. Conclusions: Polyphenol-rich plant-based additives, particularly blackcurrant pomace and extracts from *M. officinalis* and *C. asiatica*, markedly improve the antioxidant capacity, nutritional value, and sensory appeal of myofibrillar protein-based food gels. These findings support their potential application in the development of functional food products tailored to consumer expectations.

## 1. Introduction

The continued progress in research on functional food products has always been associated with their quality essential nutrients and health-promoting benefits [1]. Incorporating bioactive compounds from plant-based sources contributes to enriching the functionality of food matrices [2]. Specifically, bioactive compounds from plant-based sources are increasingly being established to have high promise in reducing oxidative stress, modulating inflammation, and lowering health risks associated with chronic diseases [3,4,5,6]. Moreover, protein-rich foods are increasingly shown to have high nutritional value with the capacity to sustain muscle function/mass [7,8].Growing interest has been observed in myofibrillar proteins (MPs), known for being primarily derived from muscle tissue. When added to food systems, MP would further activate the food system’s functionality by strengthening the desirable textural, emulsifying, and water-binding properties [9,10]. Their digestibility/amino acid profile makes MPs a valuable candidate for dietary formulations, particularly for proactive populations seeking muscle growth and maintenance [11,12,13]. Specific to the meat industry, the extraction of myofibrillar proteins from raw food materials, like those of poultry muscles, should be an added-value by-product(s) [14].

Typical examples of plant-based foods include fruits, vegetables, herbs, and spices—well known to comprise health-promoting bioactive compounds like antioxidants, phenolic compounds, and polyphenols Abd El-Hack 2023, Lang et al. 2024, [15,16]. Health-promoting bioactive compounds can help mitigate oxidative stress, with protective capacities in unhealthy situations, e.g., cardiovascular conditions, certain cancers, etc. [4,6,17]. For instance, blackcurrant extracts (juice/pomace) have established levels of anthocyanins and polyphenols, which significantly strengthen the antioxidant efficacy of emergent byproducts [18,19]. Another example is the nutritional value of *Centella asiatica*, typified by minerals and vitamins able to help elevate the overall health situation of consumers. When incorporated into animal food products, however, such health-promoting bioactive compounds could fortify the functional/nutritional stability of this emergent food matrix [2]. Moreover, the interactive capacity of health-promoting bioactive compounds obtained from plant-based sources with proteins is not a new phenomenon [20,21]. Through covalent and noncovalent bonds, which modify the (protein) structure (by aggregation, functional groups, and structure solubility/stability), the interaction of bioactive compounds with protein could result in physicochemical/functional variations [9,22,23]. Thus, myofibrillar proteins possess both a complex dynamic three-dimensional structure and physicochemical/emulsifying capabilities [14,22,23]. Further, polyphenol–protein interaction(s) confer a certain degree of bioavailability, functional, solubility, and stability implications, demonstrable by its effectiveness/efficacy in altering proteins, as well as its ability to bring about useful health/nutritional benefits [14,22]. However, as the pathways underscoring such (above-mentioned) interactions continue to be debated, there is the need to learn more about the governing principles of such interactions, especially if the development of functional food products that optimize both health/nutritional benefits and sensory attributes is to be fully realized.

As a rich source of essential amino acids, chicken protein serves as an effective dietary protein. Additionally, it has potential as a natural antioxidant source for human consumption given its well-balanced amino acid profile, extensive availability, and beneficial nutritional properties [24,25]. The valorization of myofibrillar proteins from poultry muscles could contribute to waste reduction and sustainability within the meat industry [21]. Poultry meat that is not classified by consumers as culinary due to defects such as pink discoloration of raw and cooked meat, bone darkening, red/bloody discoloration, white striping, wooden breast, spaghetti meat, or a pale, soft, and exudative breast meat appearance can serve as a valuable raw material for muscle protein isolation [26]. Utilizing such meat in protein extraction processes can contribute to minimizing raw material losses and increasing the efficiency of poultry processing. Increases in consumer awareness push the food industry to steadily seek the use of alternatives to chemical preservatives, especially those of natural sources, which positions plant-derived bioactives as promising for their combination with animal food products [13,14,23]. Incorporating a model myofibrillar protein-based approach with plant-based additives could help to enhance the nutritional value and sensorial quality of emergent food product(s). Creating a functional food product, particularly via the addition of plant-based additives, could not only satisfy the dietary protein requirements but also deliver enhanced health benefits/consumer appeal. It can be hypothesized that enriching myofibrillar proteins with plant-based additives could elevate both functionality and nutritional values, and by analyzing such aspects, one could possibly elucidate how the component ingredients are instrumental in synergistically enhancing the overall emerging product quality. To supplement existing information, therefore, the current study investigated the antioxidant, polyphenol, physical, and sensory changes in myofibrillar protein gels with polyphenol-rich plant-based additives. This study was conducted by preparing myofibrillar protein gels enriched with polyphenol-rich plant-based additives, including blackcurrant juice, blackcurrant pomace, *Melissa officinalis* extract, and *Centella asiatica* extract. The experimental design involved the extraction and standardization of myofibrillar proteins, followed by their incorporation with selected plant-derived bioactives. The physicochemical properties, antioxidant capacity, and polyphenol content of the resulting protein gels were assessed using instrumental analyses. The structural modifications and functional stability of the protein matrices were characterized to determine the impact of plant additives on protein interactions. Sensory evaluation was performed to assess consumer-relevant attributes, ensuring a comprehensive understanding of both functional and sensory implications. The anticipated findings hold significant application potential in the development of food products with tailored characteristics designed for specific consumer groups. This could be particularly relevant for individuals with increased nutritional requirements who seek a higher protein intake in their diet. Consequently, the results may contribute to advancing specialized food products for the elderly, children, and athletes, potentially enhancing their overall well-being and health.

## 2. Materials and Methods

### 2.1. Chemicals and Reagents

The reagents used for myofibrillar protein isolation were sourced as follows: KCl (100 mM) and EDTA (1 mM) were purchased from Sigma-Aldrich Chemie GmbH, Schnelldorf, Germany, phosphate buffer (25 mM) and NaCl solution (0.1 M) were supplied by Pol-Aura, Dywity, Poland, and Hydrochloric Acid (0.1 N) was procured from Chem-Pur, Piekary Śląskie, Poland. Plant-based additives included *C. asiatica* extract (2%, Water-Ethanol 1:1) from Slavia Guru, Iława, Poland, and *Melissa officinalis* extract (2%, Water-Ethanol 1:1) from Phytopharm, Nowe Miasto nad Wartą, Poland. Blackcurrant (*Ribes nigrum*) juice (100% NFC, pasteurized) and blackcurrant pomace (dried) were both provided by Oleofarm, Wroclaw, Poland. Ethanol (96%) was sourced from Avantor Performance Materials Poland S.A., Gliwice, Poland. Reagents for antioxidant activity assays were also procured from various suppliers. ABTS solution (7 mM), DPPH solution (0.3 mM), Trolox standard, and TPTZ (2,4,6-Tripyridyl-s-Triazine, 10 mM) were obtained from Sigma-Aldrich Chemie GmbH, Germany. Potassium persulfate (K_2_S_2_O_8_, 2.45 mM) was supplied by Pol-Aura, Poland, and Ferric Chloride (FeCl_3_, 20 mM) by Chem-Pur, Poland. Methanol (HPLC-grade) and acetonitrile used for LC/MS and UPLC analysis were purchased from Merck, Darmstadt, Germany. Standards for bioactive compound identification in myofibrillar protein samples, including delphinidin-3-O-glucoside, cyanidin-3-O-rutinoside, (−)-epicatechin, (+)-catechin, procyanidin B1, epigallocatechin, quercetin-3-O-glucoside, quercetin-3,4′-di-O-glucoside, kaempferol-3-O-glucoside, and myricetin-3-O-glucoside, were sourced from Extrasynthese, Lyon Nord, France. Chlorogenic and neochlorogenic acids were provided by TRANS MIT GmbH, Giessen, Germany. All other reagents not mentioned were made readily available by certified commercial retailers. Additionally, all chemicals and reagents were of analytical grade/standard.

### 2.2. Isolation of Myofibrillar Protein

**Figure 1:**
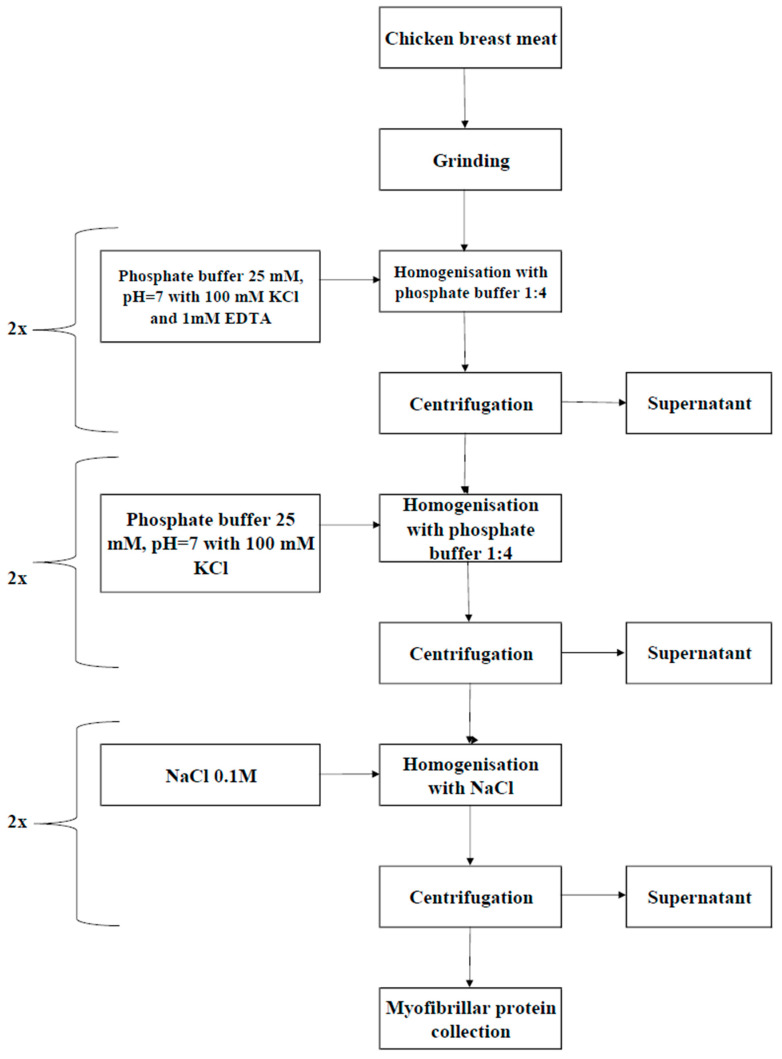
The schematic overview of the myofibrillar protein (MP) isolation process.

The procedure to isolate myofibrillar protein (MP) from chicken breast meat was largely modified from the method of Olson et al. (1976) [27] with some modifications, and the schematic overview is depicted in Figure 1. The chicken breast meat was purchased from a certified wholesale poultry farm in Wroclaw, Poland. The isolation process standard involved the homogenization of chicken breast meat muscle tissue at a 1:4 (*v*/*w*) ratio using phosphate buffer (pH 7.0, 25 mM) comprising 100 mM KCl and 1 mM EDTA. Subsequently, the homogenized sample was centrifuged at 2000× *g*, which allowed the formation of the protein sol layer. The myofibrils were then resuspended in the phosphate buffer (pH 7.0) and filtered through a polyethylene 20-mesh sieve (0.9 mm) to remove the connective tissue. After another centrifugation at 2000× *g*, the myofibril puree was washed twice with buffer (without the addition of EDTA) and filtered at each instance, followed by another centrifugation at 12,000× *g*. Then, the myofibrils were washed again using 0.1 M NaCl solution at a 1:4 ratio, followed by final centrifugation at 9000× *g*, as modified from the method of Liu and Xiong (1996) [12]. Post-washing, the pH level of the myofibrillar protein increased to 6.0 using 0.1 N HCl. The protein concentrations were obtained from myofibril suspensions following the Kjeldahl method [28]. The protein levels in basic sols and suspensions were standardized to a 50 mg/g (5%) concentration to obtain the most compact protein gels [29].

### 2.3. Preparation of Myofibrillar Protein Gels Supplemented with Plant-Based Additives

**Figure 2:**
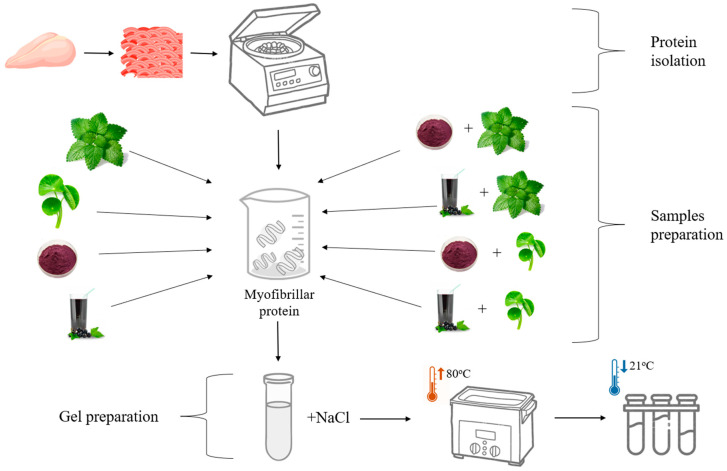
Major preparation stages of myofibrillar protein gels supplemented with different plant-based additives.

The main steps followed to prepare the myofibrillar protein gels supplemented with plant-based additives are depicted in Figure 2. The 5% myofibrillar protein solution supplemented with polyphenol-rich plant-based additives comprised the following: (a) 2% water–ethanol (1:1) extract of *C. asiatica* (Slavia Guru, Iława, Poland); (b) 2% water–ethanol (1:1) extract of *M. officinalis* (Phytopharm, Nowe Miasto nad Wartą, Poland); (c) 2% pressed blackcurrant (*Ribes nigrum*) 100% NFC (not from concentrate) pasteurized juice (Oleofarm, Wrocław, Poland); and (d) 2% dried pomace derived from blackcurrant juice extraction. To further fortify the samples for polyphenol richness, a mix of herbal and fruit additives were incorporated to achieve the 2% concentrations (the ratio of herbs extracts to pomace or juice was 1:1). All polyphenol-rich plant-derived additives were purchased from certified retailers/suppliers in Poland to ensure their quality and consistency. The control sample comprised myofibrillar proteins with no additives. The concentration of plant additives was selected based on preliminary studies conducted for *Melissa officinalis* and was applied uniformly across all raw materials to ensure consistent experimental conditions, facilitating a more reliable comparison of results.

Specifically, to prepare the myofibrillar protein gels, we followed the method described by Sulaiman et al. (2024) [13] with some modifications. The emergent myofibrillar protein sols with a 5% concentration (50 mg protein/g) were placed in 15 mm diameter polyethylene containers with a small amount of NaCl added, which adjusted the final concentration to 0.6 M. The emergent myofibrillar protein sols were then submitted to water bath thermal treatment (at ~80 °C, ensuring the complete destruction of pathogens and guaranteeing the safety of the product for consumption and consumer analysis, HACCP) until the core temperature at its geometric center reached ~70 °C, which allowed the formation of (myofibrillar protein) gels that were subsequently cooled at an ambient temperature (~25 °C) to ready them for analytical measurements. For emphasis, to prepare the myofibrillar protein gel of the current study, we employed both thermal processing conditions and little addition of NaCl, which helped to sustain the structural integrity of the myofibrillar protein gel to minimize syneresis post-cooling.

### 2.4. Analytical Methods

#### 2.4.1. Antioxidant Attributes

##### Determination of 2,2′-Azinobis(3-ethylbenzothiazoline-6-sulfonate) (ABTS+) Radical Scavenging Activity

The determination of ABTS+ radical scavenging activity followed the method described by Jeong et al. (2024) [30] with some modifications. The ABTS+ radicals were generated as 7 mM ABTS stock solutions combined with 2.45 mM potassium persulfate (K_2_S_2_O_8_), which were subsequently incubated in the dark for 16 h at 25 °C. After incubation, 990 μL of the emergent ABTS+ solution was combined with 10 μL of the supernatant containing the myofibrillar protein gel and subsequently incubated at room temperature (~25 °C) for 6 min. The control involved 990 μL of the ABTS+ solution mixed with 10 μL of 70% ethanol. The absorbance was spectrophotometrically measured at 734 nm (GENESYS™ 180, UV-VIS Spectrophotometer, ThermoFisher Scientific Inc., Waltham, MA, USA). The ABTS+ radical scavenging activity was expressed in terms of mM Trolox equivalents.

##### Determination of 1,1-Diphenyl-2-picrylhydrazyl (DPPH) Radical Scavenging Activity

The determination of DPPH radical scavenging activity followed the method described by Gulcin and Alwasel (2023) [31] with some modifications. Briefly, 20 μL of the supernatant from the already-prepared myofibrillar protein gels was thoroughly mixed with 200 μL of the 0.3 mM ethanolic DPPH solution and vortexed for 1 min. After keeping the mixture in the dark at ambient temperature (~25 °C) for 30 min, the absorbance was spectrophotometrically measured at 517 nm (GENESYS™ 180, UV-VIS Spectrophotometer, ThermoFisher Scientific Inc., Waltham, MA, USA), wherein the blank served as a reference. The DPPH radical scavenging activity was expressed in terms of mM Trolox equivalents.

##### Determination of Ferric-Reducing Antioxidant Power (FRAP) Assay

The determination of the FRAP assay followed the method described by Kalpana et al. (2016) [32] with some modifications. The ethanol extracts of myofibrillar protein gels were prepared using 70% ethanol. The FRAP reagent comprised 10 mM 2,4,6-tripyridyl-s-triazine (TPTZ), 20 mM ferric chloride, and 300 mM sodium acetate buffer (pH 3.6), mixed in a ratio of 1:1:10 (*v*:*v*:*v*) and subsequently incubated for 30 min at 37 °C. The control comprised 3 mL of the FRAP reagent combined with 1 mL of ethanol. The absorbance was spectrophotometrically measured at 593 nm (GENESYS™ 180, UV-VIS Spectrophotometer, ThermoFisher Scientific Inc., Waltham, MA, USA), and FRAP values were expressed in terms of mM/dm^3^.

#### 2.4.2. Determination of Polyphenolic Compounds by the LC-MS-PDA-Q/TOF and UPLC-PDA Methods

The determination of polyphenol content was performed using the method described by Wojdyło et al. (2021) [33] with some modifications specific to myofibrillar protein gels. This involved the use of the Acquity UPLC system (Waters Corp., Milford, MA, USA), which operated a photodiode, fluorescence detector, and mass detector/spectrometer (Waters, Manchester, UK). Comparing the absorbance values (520 nm for anthocyanins, 360 nm for flavonols, 320 nm for phenolic acid, and 280 nm for flavan-3-ols) of the pure standards with the obtained results helped to ascertain the identification of polyphenolic compounds in the analyzed samples. Quantification was performed using external calibration curves of reference polyphenols based on structural similarities. The results of the polyphenolic compounds are expressed as mg/polyphenols per 100 g, focusing on compounds like flavonols, flavan-3-ols, and phenolic acids.

#### 2.4.3. Physical Attributes

##### Determination of pH

The determination of pH followed the method described by Wojdyło et al. (2021) [33] with some modifications specific to myofibrillar protein gel samples. Samples of about 5 g and 45 mL of distilled water were mixed at 10,000 rpm for 1 min by a homogenizer (PH91, SMT Chiba, Chiba, Japan). Subsequently, the pH measurement was performed using a Schott Handylab 11 pH-meter equipped with a BlueLine 31 Rx glass electrode (Xylem Analytics Germany Sales GmbH & Co. KG, Mainz, Germany), and calibrations were performed using buffer solutions (pH 4.0, 7.0, and 9.0).

##### Determination of CIE L*a*b* Color

Color measurements were performed on the myofibrillar protein sols before and after gelation. This involved the use of the Minolta CR-40 reflective colorimeter (Konica Minolta Sensing Americas, Inc., Ramsey, NJ, USA), which operated the CIE L*a*b* color system. Prior to use, the colorimeter was calibrated by black and, thereafter, white reference standards. The operational features of the colorimeter were as follows: (a) D65 illumination (daylight, 6500 K); (b) standard observer at 10°; (c) 12 mm diameter aperture; and (d) spectral range between 360 and 740 nm. Before the color measurements, the samples were first taken out of refrigeration and allowed to equilibrate at an ambient temperature (~25 °C) for 15 min. Next, the samples were sliced transversely with a 4 cm diameter and immediately placed on a freshly exposed glass surface. Immediately after, the CIE Lab* system operated and produced three color parameters: L* (lightness), a* (red–green), and b* (yellow–blue).

##### Determination of Hardness and Cohesiveness

The hardness and cohesiveness of the already-prepared myofibrillar gels placed in cylindrical shapes (approx. 15 mm in height) were determined by instrumental texture measurements. The Zwick-Roell Universal Testing Machine (Zwick-Roell GmBH & Co. KG, Ulm, Germany) was employed, which operated a double compression test of 75% deformation and a crosshead speed of 30 mm/min. From the force–displacement curve, the hardness (N) was reported as the maximum force at the first compression, whereas the cohesiveness (%) was reported as the ratio of work performed at the second compression compared to the first compression. Specifically, both cohesiveness and hardness reflect the mechanical attributes of myofibrillar gels.

##### Determination of Calorific (Energy) Value

The calorific value analysis was performed using the method described by Hopper et al. (2024) [34] with some modifications specific to the already prepared myofibrillar gels. This involved the use of a C200 calorimeter affixed with an RC 2 basic recirculation cooler (IKA, Wilmington, NC, USA). The samples of already-prepared myofibrillar gels (1 g) were placed in a crucible and combusted by an oxygen-filled bomb calorimeter under 35 bar pressure. Adhering to the operational manual of the manufacturer, the bomb was immersed in a quantified amount of water, with some heat being released during combustion, which occurred as the temperature of the water increased. The calorific value was calculated based on the increased temperature alongside the heat capacity. Further, some corrections were made for the ignition wire. The final calorific (energy) value was expressed in kilocalories per gram (kcal/100 g).

##### Determination of Dry Matter

Dry matter was determined using the AOAC method 934.06 [28] with slight modifications specific to the already-prepared myofibrillar gels. The already-prepared myofibrillar gel, initially pre-dried in a water bath, was weighed using (name of equipment, location, country) and submitted to oven drying (Memmert UFE-500, Memmert GmbH + Co.KG, 91126 Schwabach, Germany) operating by forced-air circulation at ~105 °C until a constant mass was achieved. Dry matter (%), accurate to 0.01%, represented the relative mass comparison of the post- and pre-dried sample(s).

##### Determination of Ash

Ash content was determined using the AOAC method 923.03 [28] typified by the mass difference technique. The already-prepared myofibrillar gel (~2 g), placed in pre-weighed porcelain crucibles and initially carbonized, was incinerated (time = 16 h; temperature = 550 °C) using a muffle furnace. Subsequently, emergent samples were cooled to room temperature in a desiccator, followed by the reweighing of crucibles. Ash content was determined by the mass differences before and after incineration.

#### 2.4.4. Sensory Attributes

The sensory evaluation followed the method described by Civille, Carr, and Osdoba (2024) [35] with some modifications to the already-prepared myofibrillar gels at least two hours after the sensory evaluators’ last meals. The sensory evaluation was specifically conducted in an air-conditioned (sensory) facility equipped with booths and sinks for oral rinsing. Eight trained sensory evaluators provided verbal consent prior to their voluntary participation, with the assurance that no names or genders were collected to ensure their privacy, and they were familiarized with the product’s intensity (e.g., pore number, texture firmness, attractiveness, color intensity, and odor perception) and sensory (e.g., color, odor, texture, structure, and overall impression) attributes. The sensory evaluators sampled the myofibrillar protein gels (weight = 15 g; temperature = 7 °C) placed in coded containers. During the sensory session, the evaluators assessed a total of 9 gel and control samples. The first five types of gel samples were assessed. A 30 min break followed to prevent fatigue. Mouth rinsing was carried out with clean warm water [35]. Thereafter, the remaining five were assessed. For emphasis, two evaluation methods were considered, namely consumer preference and sensory profile. Consumer preference comprised a 1–5 scale rating for color, consistency, structure, and taste. The sensory profile comprised a 1–9 scale rating for aroma intensity, color intensity, consistency, porosity, and taste [36]. Mean values and standard deviations were recorded based on panelist ratings.

### 2.5. Statistical Analysis

The generated data were submitted to a one-way analysis of variance (ANOVA) and presented in terms of means ± standard error (SEM) unless otherwise stated. Further, Duncan’s test was applied to resolve the means. Unless otherwise stated, experimental measurements were performed in triplicate. The probability level has been set at *p* < 0.05 to ascertain statistical significance. To examine the relationships between selected parameters, Pearson’s correlation analyses were conducted. All statistical analyses, including ANOVA and correlation tests, were performed using Statistica 13.0 software (StatSoft Inc., New York, NY, USA).

## 3. Results and Discussion

### 3.1. Antioxidant Changes

The antioxidant changes were largely statically significant (*p* < 0.05) across myofibrillar protein sols with different plant-based additives, which were depicted by determinations of ABTS, DPPH, and FRAP, as shown in Table 1. The controls with the lowest antioxidant values, specifically 0.17 ± 0.01 mM Totox for ABTS, 0.01 ± 0.04 mM Totox for DPPH, and 1.93 ± 0.05 mM/dm^3^ for FRAP, were suggestive of limited radical-scavenging/reducing capacity. Clearly, adding plant extracts could enhance the antioxidant capacities, distinctly more by ABTS and FRAP than DPPH. For instance, the ABTS and FRAP values of the *M. officinalis* extract with myofibrillar protein (ABTS = 2.49 ± 0.05 mM Trolox; FRAP = 4.62 ± 0.08 mM/dm^3^) seemed comparable to those of blackcurrant pomace (ABTS = 2.35 ± 0.05 mM Trolox; FRAP = 4.68 ± 0.14 mM/dm^3^). This result suggests the somewhat mild antioxidant impact of blackcurrant juice/pomace in the current work. Further, the DPPH results suggested increased radical neutralization, specifically by blackcurrant pomace (0.13 ± 0.01 mM Trolox), which appeared to resemble *M. officinalis* combined with pomace (0.13 ± 0.00 mM Trolox). Shakeri et al. (2016) [37] understood the high polyphenolic contents of *M. officinalis* extract, as typified by rosmarinic acid, to contribute to strengthening its antioxidant activity. On the other hand, *C. asiatica* extract showed moderate increases in ABTS (from 2.14 ± 0.02 to 2.44 ± 0.02 mM Trolox) and FRAP values (from 3.24 ± 0.40 mM/dm3 to 3.42 ± 0.31 mM/dm^3^), suggestive of somewhat effective yet less potent antioxidant activity compared to either blackcurrant pomace or *M. officinalis* extract. The earlier study of James and Dubery (2009) [38] described the promising antioxidant profile of *C. asiatica* extract, particularly how it was associated with triterpenoids like asiatic acid and madecassic acid, which corroborates the significant ABTS and FRAP values obtained in the current work. Moreover, adequately integrating *C. asiatica* extract into a given myofibrillar protein system might confer promising health benefits, especially in terms of enhancing antioxidant capacities. Moreover, adding blackcurrant juice in the current work seemed to enhance the antioxidant activity but to a lesser extent. In the context of comparing the emerging ABTS, DPPH, and FRAP values, Dulf et al. (2016) [39] revealed that blackcurrant pomace/juice could influence the antioxidant activity of a given myofibrillar protein system. Earlier work like that of Määttä-Riihinen et al. (2004) [40] indicated that the antioxidant potential of blackcurrant could enhance the color aspects of food products. Moreover, hydroxyl groups donate hydrogen atoms or electrons, neutralizing reactive oxygen species and preventing oxidative damage [41]. Interactions with proteins occur via hydrogen bonding, hydrophobic forces, and covalent modifications, leading to structural stabilization and enhanced resistance to oxidation [42].

### 3.2. Polyphenolic Changes

Polyphenolic compounds were identified in different myofibrillar protein (MP) samples with plant additives, as shown in Table 2. Interestingly, no polyphenol contents were detected in the control of the current work, which resembled those of blackcurrant juice. However, blackcurrant pomace samples showed a promising range of polyphenols, e.g., flavan-3-ols like procyanidins (B1, A2), flavonols like myricetin and quercetin derivatives, and anthocyanins (delphinidin and cyanidin derivatives). Even when bioactive compounds in juices have lower concentrations, they still reflect the plant-additive contributions of the overall polyphenolic contents [43]. Further, Table 2 shows some specific polyphenols in *M. officinalis* and *C. asiatica* extracts. For example, *M. officinalis* showed (+)-catechin, caftaric acid, and rosmarinic acid, whereas *C. asiatica* showed chlorogenic acid and various caffeoyl quinic acids—all of which differ from those of blackcurrant pomace in this study. For instance, *M. officinalis* extract combined with blackcurrant pomace revealed certain polyphenols such as rosmarinic acid and procyanidins, whereas *C. asiatica* extract combined with blackcurrant pomace revealed certain polyphenols such as procyanidins and chlorogenic acids. Indeed, plant extracts combined with blackcurrant pomace could further enrich the polyphenolic profile. Polyphenols influence the antioxidant properties of proteins through direct radical scavenging, metal chelation, and structural modifications of protein molecules [44].

**Figure 3:**
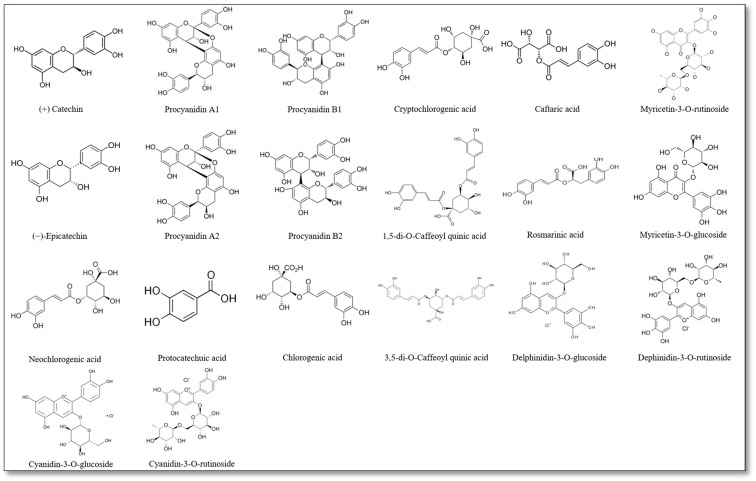
Chemical structure of phenolic compounds identified in myofibrillar protein samples with plant-based additives.

The chemical structures of phenolic compounds identified in myofibrillar protein samples supplemented with plant-based additives are depicted in Figure 3. While the bioactive compounds detected include catechins, procyanidins, chlorogenic acids, and anthocyanins, their biological activity was reflective of specific functional groups. For instance, such hydroxyl groups (-OH) in flavonoids like catechins and procyanidins possess antioxidant components that donate hydrogen atoms to neutralize free radicals. Also, such carboxyl groups (-COOH) in phenolic acids like chlorogenic and caffeic acids could contribute to the free radical scavenging processes. Further, glycosidic bonds in anthocyanins (e.g., cyanidin and delphinidin glycosides) could enhance the bioavailability/solubility of antioxidants with certain anti-inflammatory effects [45,46,47]. Moreover, the presence of these functional groups may facilitate interactions with protein side chains, leading to modifications in structural stability and gelation properties [48,49]. The formation of polyphenol–protein complexes and their resulting antioxidant properties are influenced by the mode of binding, the type of protein or polyphenol, and external conditions [50]. Polyphenols can also inhibit protein carbonylation and thiol oxidation, preserving functional groups essential for protein integrity [51]. The formation of polyphenol–protein complexes may enhance or limit antioxidant potential depending on binding affinity and accessibility to reactive species [52].

Polyphenol–protein interactions are complex and may be influenced by factors such as pH, ionic strength, and temperature [53]. Studies have shown that flavonoids, particularly catechins, can form both noncovalent and covalent bonds with proteins, altering their secondary structure and functional properties [54]. Additionally, anthocyanins have been reported to interact with myofibrillar proteins, influencing their solubility and aggregation behavior [55].

**Figure 4:**
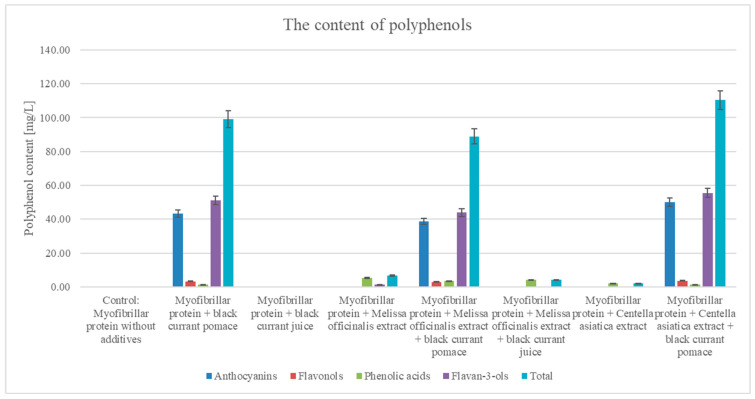
The levels of polyphenol contents in myofibrillar protein samples supplemented with plant additives.

The levels of polyphenol content across myofibrillar protein supplemented with plant-based additives are depicted in Figure 4. Specifically, different levels of polyphenolic compounds were detected, from anthocyanins, flavonols, and phenolic acids to flavan-3-ols. The control sample showed negligible polyphenol contents. In contrast, adding blackcurrant pomace increased the polyphenol content, with the latter being dominated by anthocyanins (Table 2). Further, adding blackcurrant juice also increased the polyphenol content, although this remained lower than that of pomace. Moreover, adding *M. officinalis* extract seemed to moderately increase the polyphenol content, with the latter being somewhat dominated by phenolic acids (Table 2). Typically found at lower levels, both anthocyanins and flavonols should reflect the polyphenolic profile of *M. officinalis* [56]. Nonetheless, the polyphenol content of *C. asiatica* extract seemed lower and consisted of more phenolic acids and fewer anthocyanins. It can be suggested that such constituent polyphenolic outcomes should depict the distinct (polyphenolic) profile within the myofibrillar protein matrix. Conversely, blackcurrant juice combined with either *M. officinalis* or *C. asiatica* produced lower total polyphenol content compared to those of the pomace (Figure 4). Particularly, anthocyanins seemingly dominate in blackcurrant derivatives. *M. officinalis* and *C. asiatica* extracts showed moderate polyphenol levels, which created a relatively balanced (polyphenol) profile even when combined with blackcurrant additives. While beneficial, blackcurrant juice may deliver fewer polyphenols compared to pomace [43]. In conclusion, blackcurrant pomace appears to be a stronger polyphenol-enriching candidate than juice, and even more so with the addition of plant extracts such as *M. officinalis* and *C. asiatica*.

In myofibrillar proteins, polyphenols contribute to oxidative stability by modulating redox activity and reducing the formation of secondary oxidation products, which is particularly relevant in food systems prone to lipid and protein oxidation [57]. Their effectiveness varies based on molecular structure, concentration, and environmental conditions, highlighting the need for optimized formulations to maximize their protective effects in protein-based matrices. From a nutritional perspective, polyphenols not only improve the oxidative stability of protein-based foods but may also contribute to potential health benefits, such as reducing oxidative stress and inflammation [58]. Incorporating plant extracts into protein-based food systems presents significant challenges related to solubility, texture modifications, and overall product stability [59]. Many polyphenols and bioactive compounds exhibit poor water solubility, which affects their homogeneous distribution in food matrices [60]. Limited solubility can lead to precipitation, phase separation, and reduced bioavailability, ultimately impacting their functional effectiveness [61]. One approach to improving the solubility of polyphenols in food applications is the use of organic solvents such as ethanol, which is widely recognized as a food-grade solvent. Ethanol enhances the extraction and dispersion of polyphenols, facilitating their incorporation into food systems while maintaining regulatory safety standards [62].

### 3.3. Physical Changes

The physical changes specific to color (L*, a*, and b* scales), pH, and gel strength across the myofibrillar protein samples supplemented with plant-based additives are shown in Table 3. In terms of color, the control exhibited increased lightness (L* = 76.36 ± 1.82), a slight green hue (a* = −0.92 ± 1.20), and somewhat yellowness (b* = 18.59 ± 2.06). In contrast, adding blackcurrant pomace slightly reduced the lightness (L* = 73.33 ± 2.49), increased redness (a* = 2.59 ± 1.28), and slightly decreased yellowness (b* = 15.99 ± 33.60), suggesting that the pigment concentration influences the protein color/matrix [63]. Adding *M. officinalis* extract moderately decreased lightness (L* = 70.52 ± 0.26), increased the green hue (a* = −1.75 ± 0.05), and reduced yellowness (b* = 14.03 ± 1.16), while *C. asiatica* extract alone reduced lightness (L* = 69.60 ± 3.94) and increased redness (a* = 0.16 ± 0.85) and yellowness (b* = 21.45 ± 0.74). The slight darkening effect (a decrease in the L* value) suggests the formation of protein–polyphenol complexes, which has been previously linked to the oxidation of polyphenols leading to the formation of darker-colored quinones [64]. When *C. asiatica* extract was combined with blackcurrant pomace, lightness decreased further (L* = 64.28 ± 3.25), with increases in redness (a* = 1.56 ± 3.39) and yellowness (b* = 21.73 ± 2.33). However, *C. asiatica* extract combined with blackcurrant juice appeared lighter (L* = 71.31 ± 1.98) with reduced redness (a* = 0.05 ± 1.37) and reinforced yellowness (b* = 28.20 ± 3.39). Although *M. officinalis* and *C. asiatica* extracts enhanced the yellowness of myofibrillar protein gel, the effect appeared stronger for *C. asiatica*. More so, *C. asiatica,* especially when combined with blackcurrant pomace, further intensified the yellowness of myofibrillar protein gel. The interaction of plant extracts with proteins through hydrogen bonding, hydrophobic interactions, and covalent modifications can alter the structural integrity of food products [65]. These interactions may lead to excessive protein cross-linking, changes in gelation behavior, and the formation of undesired precipitates, negatively influencing texture and water-holding capacity [66].

In terms of pH, there were increases in protein sols (pH) when adding blackcurrant pomace (pH = 6.25 ± 0.01), somewhat similar to when adding blackcurrant juice (pH = 6.23 ± 0.02), which seemed to moderate the protein system compared to the control (pH = 6.17 ± 0.01). In contrast, the pH of protein sols decreased when adding *M. officinalis* extract (pH 6.13 ± 0.01), which was slightly acidified and resembled those of either blackcurrant pomace (pH = 6.13 ± 0.01) or juice (pH = 6.13 ± 0.00), consistent with adding *C. asiatica* extract (pH = 6.14 ± 0.01) and combining blackcurrant pomace (pH = 6.37 ± 0.02) and juice (pH = 6.26 ± 0.04). Both *M. officinalis* and *C. asiatica* extracts have strong impacts on pH compared to blackcurrant pomace/juice in this study. In terms of gel strength, besides the control samples with a peak gel hardness (0.87 ± 0.28 N) indicative of a more resistant structure, some (gel hardness) reductions occurred when adding blackcurrant juice (0.42 ± 0.15 N) or pomace (0.27 ± 0.29 N). Possibly, both pomace and juice weaken the gel structure. Adding *M. officinalis* extract decreased the gel hardness (0.36 ± 0.29 N), which decreased much more when combining blackcurrant pomace (0.20 ± 0.14 N) or juice (0.28 ± 0.12 N). In contrast, adding *C. asiatica* extract increased gel hardness (0.55 ± 0.21 N), but this decreased when combining blackcurrant pomace (0.33 ± 0.25 N) or juice (0.21 ± 0.17 N). It is possible that the *C. asiatica* extract enhanced the gel structure, which could have been diminished when adding blackcurrant additives [67,68]. The formation of protein–polyphenol complexes influences both the antioxidant potential and functional properties of the modified proteins. Research has demonstrated that polyphenols can enhance various functional properties of MP. For instance, Jongberg et. al [69] found that green tea extract (GTE) at low concentrations contributed to the oxidative and textural stability of meat emulsions [70] observed that the emulsifying activity of MP improved with the addition of sage extract. [71] reported that incorporating pomegranate peel and grape seed extracts into MP-based edible films under acidic conditions led to an increase in tensile strength (TS). Additionally, [72] demonstrated that treating MP with chlorogenic acid and a Fenton-mediated oxidation system significantly enhanced its gelation properties within the range of 6 to 30 μmol/g of the phenolic compound.

**Figure 5:**
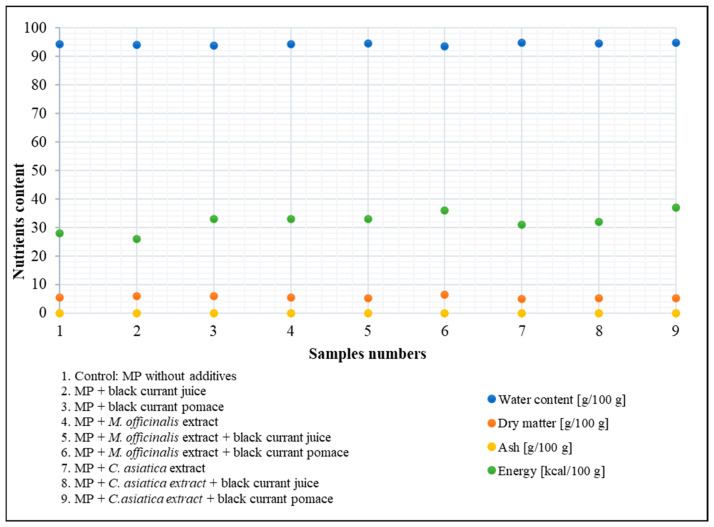
Ash, dry matter, energy, and water contents of myofibrillar protein supplemented with plant additives compared to control (the abbreviation “MP” used in sample labeling stands for myofibrillar protein).

The ash, dry matter, energy, and water contents of myofibrillar protein with plant additives compared to the control are shown in Figure 5. In terms of dry matter, the control was 5.56 g/100 g, which slightly increased by adding blackcurrant juice (5.99 g/100 g), and more so when adding pomace (6.07 g/100 g). Adding *M. officinalis* extract decreased dry matter (5.66 g/100 g), which decreased even further when combined with blackcurrant juice (5.31 g/100 g), a result that was not replicated when adding pomace (6.47 g/100 g). Adding *C. asiatica* extract slightly decreased the dry matter (5.08 g/100 g), but this was not replicated when combined with blackcurrant juice (5.31 g/100 g) and pomace (5.18 g/100 g). Water content remains inversely related to dry matter [73]. In terms of water content, there were slight changes when adding blackcurrant juice (94.01 g/100 g) and pomace (93.93 g/100 g) compared to the control (94.44 g/100 g), but this was not so for *M. officinalis* extract alone (94.34 g/100 g), even when the latter was combined with blackcurrant juice (94.69 g/100 g) or pomace (93.53 g/100 g). Interestingly, the water content increased when adding *C. asiatica* extract (94.92 g/100 g), but not much when the latter was combined with blackcurrant juice (94.69 g/100 g) and pomace (94.82 g/100 g). Ash content, which is representative of the mineral content [74], seemed consistent across all samples, ranging from 0.01 to 0.02 g/100 g, suggesting that the addition of plant-based ingredients would likely not alter the mineral composition of myofibrillar protein gels. In terms of the energy value, slight increases appeared when adding blackcurrant juice (26 kcal/100 g), with an even higher increase when adding pomace (33 kcal/100 g), despite the latter resembling the effect of *M. officinalis* extract (33 kcal/100 g), which increased when combined with blackcurrant pomace (36 kcal/100 g) and decreased when combined with blackcurrant juice (33 kcal/100 g) compared to the control (28 kcal/100 g). Although *C. asiatica* extract alone produced an energy value of 31 kcal/100 g, this slightly increased when combined with blackcurrant juice (32 kcal/100 g) and even more so when combined with pomace (37 kcal/100 g).

### 3.4. Sensory Changes

**Figure 6:**
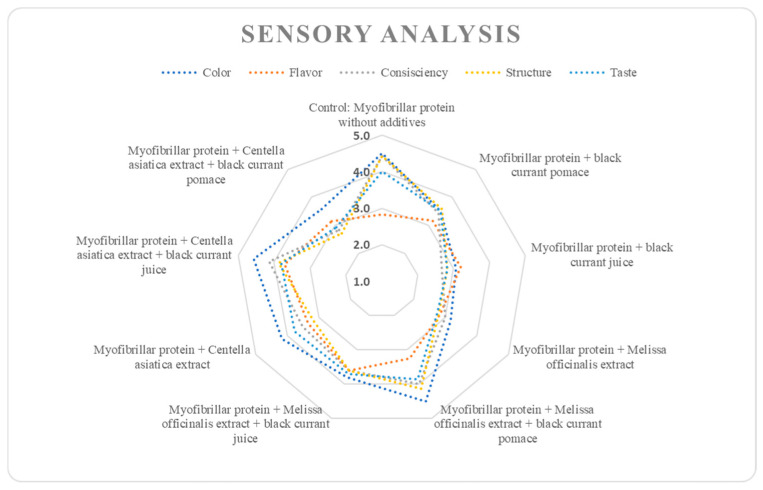
Sensory display of myofibrillar protein gels supplemented with plant additives.

**Figure 7:**
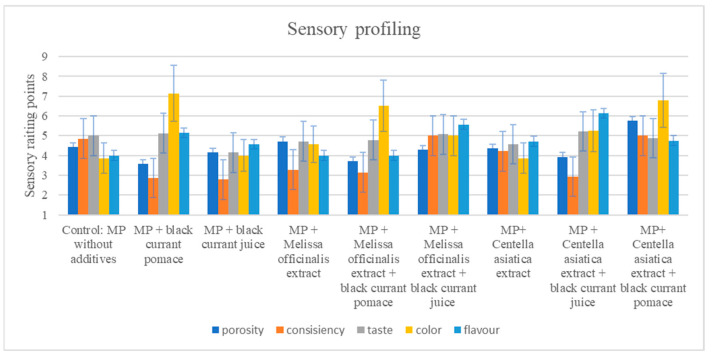
Sensory profile of myofibrillar protein gels supplemented with plant additives (the abbreviation “MP” used in sample labeling stands for myofibrillar protein).

To decipher the impact of various plant additives on the sensory aspects of myofibrillar protein gels, a trained (sensory) panel performed consumer preference testing and profiling, as shown in Table 4 and Figure 6 and Figure 7. As shown in Table 4, the relatively high color score of the control (4.50 ± 0.84) decreased with the addition of blackcurrant pomace (3.50 ± 0.84) and was much lower with juice (3.07 ± 0.84), but this was not so when *M. officinalis* extract was combined with blackcurrant pomace (4.50 ± 0.84). However, the color score of *C. asiatica* extract combined with blackcurrant juice (4.57 ± 0.79) seemed to be favored by the sensory panel. The range in flavor scores from the control (2.83 ± 1.33) to the combination of *C. asiatica* extract and blackcurrant juice (3.71 ± 0.76) suggests minimal (flavor) effects. When adequately introduced, *C. asiatica* had a slightly bitter flavor, which might help to enhance a product’s acceptability when employed in appropriate quantities [75]. Without compromising the overall product quality, the enriched polyphenol nature of blackcurrants, largely impacted by its intense red–purple color, would be effective in enhancing nutritional/functional aspects and consumer visual acceptance/appeal [76].

The peak consistency score of the control (4.43 ± 0.79) showed reduced cohesive strength with blackcurrant juice (2.67 ± 1.03), but this was not the case when *M. officinalis* extract was combined with blackcurrant pomace (4.00 ± 1.00) or *C. asiatica* extract was combined with blackcurrant juice (4.14 ± 0.69). The peak structure score of the control (4.43 ± 0.79) showed reduced integrity with blackcurrant juice (2.83 ± 0.98), but this was not the case when *M. officinalis* extract was combined with blackcurrant pomace (4.14 ± 0.69) or *C. asiatica* extract was combined with blackcurrant juice (3.88 ± 0.99). The peak taste score of the control (4.00 ± 0.58) showed reduced intensity for blackcurrant juice (2.83 ± 0.52), but this was not the case when *M. officinalis* extract was combined with blackcurrant pomace (3.86 ± 0.85) or *C. asiatica* extract was combined with blackcurrant juice (3.81 ± 0.92). *M. officinalis* extract, by contributing to the aroma and flavor of food products, could provide useful sensory and health benefits [77].

While contributing to oxidative stability, polyphenols may also introduce bitter or astringent flavors for consumers, potentially reducing their sensory acceptability. However, given the diverse molecular structures/binding mechanisms involved, pinpointing the specific sensory effects, as well as linking them to their molecular impact, remains a complex challenge [78]. These interactions can lead to either strengthening or weakening of the protein gel matrix, depending on the specific type and concentration of polyphenols involved [50]. Notwithstanding, noncovalent interactions between food macromolecules, such as proteins and polysaccharides, and polyphenols could noticeably influence the sensory characteristics of food. Figure 6 and Figure 7 reveal the sensorial display and profiles of myofibrillar gels with plant additives, respectively, specifically the (sensorial) dynamics when panels observed the myofibrillar protein gels. For instance, the addition of blackcurrant juice might negate the enhanced sensorial consistency, structure, and taste resulting from the *C. asiatica* extract. Moreover, the sensory quality effect might be less pronounced in blackcurrant pomace compared to juice, but this may not be so when combined with *M. officinalis* extract, which seemed to restore (sensory) color/texture. Furthermore, the addition of blackcurrant pomace not only boosted antioxidant activity but also impacted sensory color and texture. Functionally, the strategic utilization of such plant-based additives could moderate the sensory aspects of the myofibrillar protein gels used in the current study.

### 3.5. Relationships Between Antioxidant, Polyphenolic, Physicochemical, and Sensory Attributes

**Figure 8:**
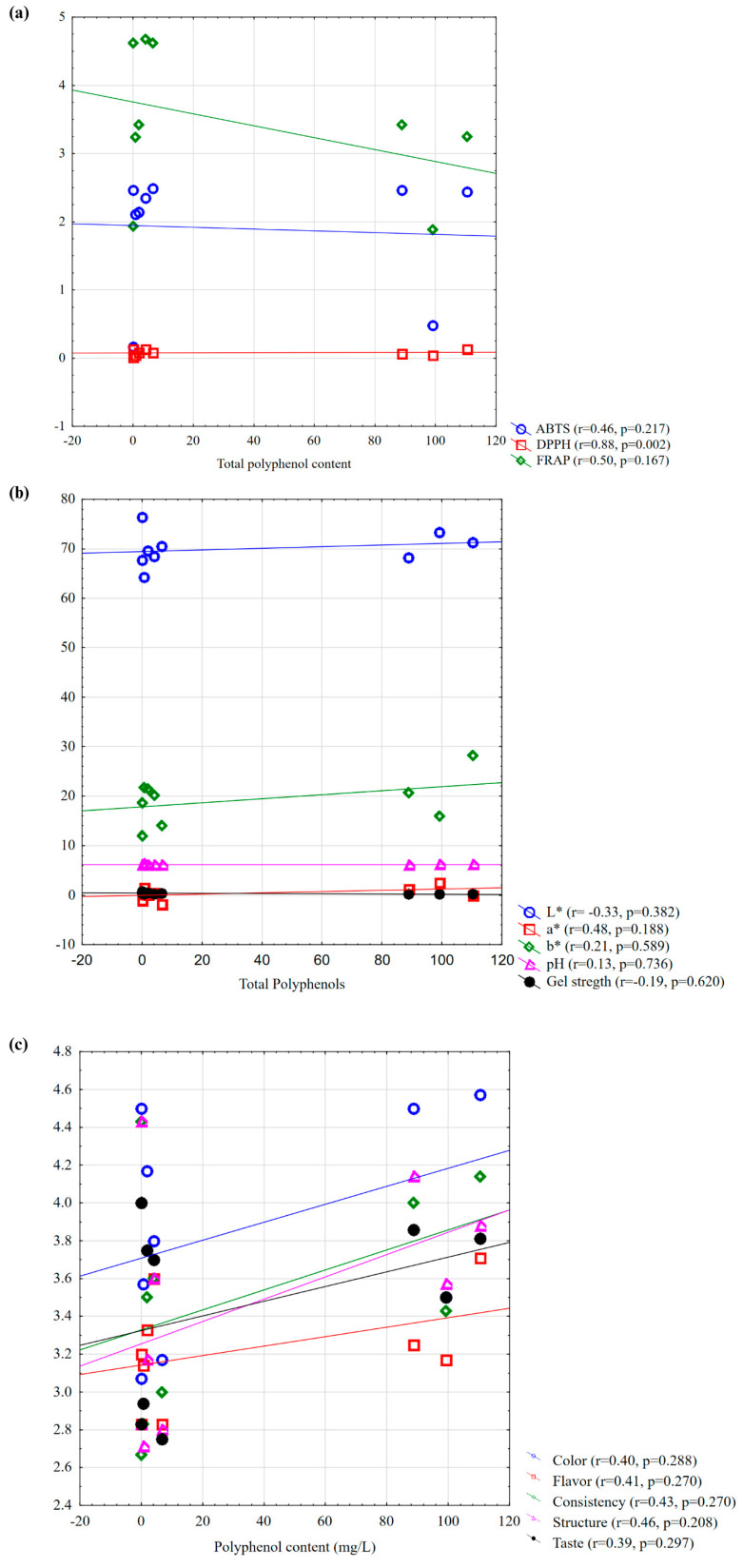
Relationships between total polyphenol content and selected physicochemical and sensory parameters. (**a**) Correlation between total polyphenol content and antioxidant activity indicators (ABTS, DPPH, and FRAP); (**b**) correlation between total polyphenol content and physicochemical parameters (L*, a*, b*, pH, and gel strength); (**c**) correlation between total polyphenol content and sensory parameters (color, taste, aroma, consistency, and structure).

Given the antioxidant, physicochemical, polyphenolic, and sensory outcomes of protein-based food products obtained in this work, correlation tests were performed to elucidate their potential relationships. Figure 8 depicts the relationships between total polyphenols and select physicochemical and sensory parameters. The results revealed the strongest relationship in the DPPH assay (r = 0.88, *p* = 0.002) (Figure 8a), suggesting a pronounced radical-scavenging ability of polyphenols in this specific myofibrillar protein-based system. Further, the ABTS assay exhibited a moderate but statistically non-significant positive correlation (r = 0.46, *p* = 0.217), indicating some potential yet inconsistent antioxidant effects. Additionally, the FRAP assay exhibited a moderate negative trend (r = −0.50, *p* = 0.167), suggesting that higher polyphenol content might not necessarily enhance the reducing power in this specific myofibrillar protein-based system. In agreement with this finding is the previous report of Prior et al. (2005) [20], which noted that different polyphenol structures exhibit varying affinities for radical species. The negative trend observed in the FRAP assay seems to also agree with the recent findings of Xu et al. (2021) [79], who suggested that some polyphenols, particularly anthocyanins, might not contribute effectively to reducing power due to their structural instability at certain pH levels.

Anthocyanins, flavonols, and tannins have been shown to interact with proteins, influencing color stability and pigment retention in food matrices [80,81]. The relationship between polyphenol content and physicochemical properties suggests a potential effect on color but a limited influence on other parameters (Figure 8b). Increased polyphenol content was associated with a slight decrease in lightness (L*, r = −0.33) and minor shifts toward red (a*, r = 0.48) and yellow hues (b*, r = 0.21). However, no significant correlations were observed for pH (r = 0.13) or gel strength (r = −0.19), indicating that under the tested conditions, the addition of polyphenol did not markedly affect these properties. However, the absence of a significant correlation between pH and gel strength is in contrast with certain previous reports indicating that polyphenols can influence protein gelation by promoting or inhibiting protein aggregation [82]. Differences in polyphenol–protein interaction mechanisms, polyphenol concentrations, or the specific structural properties of the proteins studied might cause such discrepancies.

Herein, the polyphenols were found to moderately modify the perception of structure and consistency in myofibrillar protein gels (r = 0.46 and r = 0.43, respectively), likely due to the protein–polyphenol interactions affecting gel network formation and water-holding capacity (Figure 8c). The correlation with color perception seemed moderate (r = 0.40), suggesting that while polyphenols, particularly anthocyanins, could enhance color intensity, other factors might also contribute to visual perception. The weakest correlations were observed for taste (r = 0.39) and aroma (r = 0.41), reflecting the complex role of polyphenols in sensory attributes. The correlation findings between polyphenol content and color perception further support those of Castañeda-Ovando and colleagues, who reported that anthocyanin-rich extracts could enhance visual appeal, contributing to consumer preference for naturally colored food products [83]. However, the weak correlations with taste and aroma might suggest that while polyphenols contribute to antioxidant stability, their influence on flavor appears more complex, often introducing bitterness and astringency through their interaction with salivary proteins [84,85].

## 4. Limitations of the Current Study

While this study provides significant insight into the impact of polyphenol-rich plant extracts on the physicochemical, structural, and sensory properties of myofibrillar protein gels, several limitations should be acknowledged to contextualize the findings and guide future research. One limitation of the current study is that it focuses on total polyphenol content rather than the specific contributions of individual polyphenolic compounds, making it challenging to determine their precise role in protein–polyphenol interactions, antioxidant activity, and sensory perception. Given that polyphenols exhibit diverse structures and reactivity, their interactions with proteins may vary significantly depending on their molecular characteristics. However, previous studies have demonstrated that polyphenol-rich plant extracts, rather than isolated compounds, play a significant role in food system functionality. The impact of polyphenols on protein digestibility is noteworthy, especially the modulation of enzymatic hydrolysis, which could influence bioavailability. Moreover, polyphenols could reduce protein digestion in vitro due to covalent and noncovalent interactions with proteins. Variations arising from such studies might arise from differences in the polyphenol-to-protein ratio, enzyme-to-protein ratio, digestion time, and evaluation methods [86].

The sensory evaluation was conducted using trained panelists, providing valuable insight into the descriptive attributes of polyphenol-enriched gels. However, the consumer acceptance testing herein might limit the ability to assess real-world preferences and market feasibility. Despite this limitation, trained panel evaluations are widely known to play a key role in the early stages of product development, which provides key sensory indicators that can be induced by polyphenol enrichment. The stability of polyphenol–protein interactions over time remains another critical aspect that was not assessed in this current study. Polyphenols are known to undergo oxidation, polymerization, and degradation, which may influence their antioxidant efficacy, sensory characteristics, and interactions with proteins. Given that the antioxidant activity of polyphenol–protein systems remains stable during short-term storage, the current study did not explore long-term stability assessments under different environmental conditions, which should include temperature, humidity, and light exposure, all of which can help ascertain the viability of polyphenol-enriched protein matrices.

The geographical specificity of the plant extracts used could be another limitation in the current study. The method of plant extract isolation, as well as the environmental conditions and agricultural practices, may significantly influence the polyphenol composition of the extracts, leading to variations in antioxidant activity and sensory attributes. Given the well-documented impact of soil composition, climate, and post-harvest processing on the phenolic profile of plant materials, future studies could benefit from a broader range of plant extracts derived from different geographical regions, considering different cultivation conditions and processing methods. Despite these limitations, this current study contributes to the growing body of knowledge on the functional applications of polyphenol-rich plant extracts in protein-based food systems. The current findings provide novel insights into protein–polyphenol interactions, which are crucial for optimizing the sensory and functional properties of plant-enriched protein matrices.

## 5. Conclusions

The current research highlights the multi-functional benefits that incorporating plant extracts such as blackcurrant (*Ribes nigrum*), *M. officinalis*, and *C. asiatica* could have on myofibrillar protein gel products, from antioxidant/nutritional values to sensory aspects. The addition of *M. officinalis* could significantly improve the antioxidant capacity of myofibrillar protein samples. Given the high anthocyanin levels and improved product color, blackcurrant pomace significantly increased antioxidant activity/polyphenol content. In developing functional foods with enhanced consumer appeal and nutritional value, plant-based additives remain highly promising. Indeed, we examined an innovative functional food product based on myofibrillar proteins supplemented with polyphenol-rich plant additives.

Future studies should be directed toward investigations of the oxidative stability of myofibrillar protein gels enriched with plant-based additives under different storage conditions/simulated retail environments, including microbiological shelf stability/quality over time and an exploration of the impact of packaging methods such as vacuum sealing or modified atmosphere packaging. Future studies should incorporate hedonic and affective tests to evaluate the sensory acceptability and purchase intent of consumers regarding myofibrillar protein gels, not only under different packaging methods but also under varying storage temperatures. Future research could also benefit from advanced chromatographic and spectroscopic techniques to identify and quantify individual polyphenolic compounds and elucidate their specific effects on protein-based matrices. Such research could help elucidate the molecular mechanisms underlying the covalent and noncovalent interactions with proteins, particularly regarding protein–polyphenol complex formation and the implications for food texture, stability, and sensory acceptance, as well as the impact on digestion and metabolic health. Understanding the influence of thermal processing conditions and the optimization of plant-based additive formulations on the antioxidant efficacy, nutritional stability, sensory quality, and structural integrity of myofibrillar protein gels warrants further research.

## Figures and Tables

**Table 1:** Antioxidant changes across myofibrillar protein gels supplemented with different plant-based additives.

	ABTS [mM Trolox]	DPPH [mM Trolox]	FRAP [mM/dm^3^]
Control: Myofibrillar protein without additives	0.17 ^a^ ± 0.01	0.01 ^a^ ± 0.04	1.93 ^a^ ± 0.05
Myofibrillar protein + black currant juice	0.48 ^b^ ± 0.02	0.04 ^b^ ± 0.02	1.89 ^a^ ± 0.14
Myofibrillar protein + black currant pomace	2.46 ^e^ ± 0.02	0.13 ^d^ ± 0.01	4.62 ^c^ ± 0.08
Myofibrillar protein + *M. officinalis* extract	2.49 ^e^ ± 0.05	0.08 ^c^ ± 0.04	4.62 ^c^ ± 0.08
Myofibrillar protein + *M. officinalis* extract + black currant juice	2.46 ^e^ ± 0.02	0.07 ^c^ ± 0.01	3.42 ^b^ ± 0.30
Myofibrillar protein + *M. officinalis* extract + black currant pomace	2.35 ^d^ ± 0.05	0.13 ^d^ ± 0.00	4.68 ^c^ ± 0.14
Myofibrillar protein + *C. asiatica* extract	2.14 ^c^ ± 0.02	0.08 ^c^ ± 0.01	3.42 ^b^ ± 0.31
Myofibrillar protein + *C. asiatica* extract + black currant juice	2.11 ^c^ ± 0.02	0.04 ^b^ ± 0.02	3.24 ^b^ ± 0.40
Myofibrillar protein + *C. asiatica* extract + black currant pomace	2.44 ^e^ ± 0.02	0.13 ^d^ ± 0.01	3.25 ^b^ ± 0.38
*p*	0.0000	0.0000	0.0000
SEM	0.208	0.0106	0.201

Values in the same column with different uppercase letters (a, b, c, etc.) indicate statistically significant differences between means (*p* < 0.05), determined by post hoc multiple comparison tests. Identical letters denote groups that do not differ significantly. The *p*-value (*p*) represents the significance level of the statistical test, where *p* < 0.05 indicates significant differences among samples. The standard error of the mean (SEM) reflects the precision of the mean estimate, with lower values indicating greater reliability of the measurement.

**Table 2:** Polyphenolic compounds identified in different myofibrillar protein (MP) samples supplemented with plant additives.

		Control: Myofibrillar protein without additives	Myofibrillar protein + *M. officinalis* extract	Myofibrillar protein + *C. asiatica* extract	Myofibrillar protein + black currant juice	Myofibrillar protein + black currant pomace	Myofibrillar protein + M.officinalis extract + black currant pomace	Myofibrillar protein + *M. officinalis* extract + black currant juice	Myofibrillar protein + *C. asiatica* extract + black currant juice	Myofibrillar protein + *C. asiatica* extract + black currant pomace
flavan-3-ols	Procyanidin B1	−	−	−	−	+	+	−	−	+
	(−)-Epigallocatechin	−	−	−	−	−	−	−	−	−
	(+)Catechin	−	+	−	−	+	+	−	−	+
	Procyanidin B2	−	−	−	−	+	−	−	−	+
	(−)-Epicatechin	−	−	−	−	−	−	−	−	+
	Procyanidin C1	−	−	−	−	−	−	−	−	−
	Procyanidin A1	−	−	−	−	−	−	−	−	+
	Procyanidin A2	−	−	−	−	+	−	−	−	−
phenolic acids	Neochlorogenic acid	−	−	−	−	+	+	−	−	+
	Protocatechuic acid	−	−	−	−	+	+	−	−	−
	Chlorogenic acid	−	−	+	−	+	+	−	+	+
	Caftaric acid	−	+	−	−	−	−	+	−	−
	Syringic acid	−	−	−	−	−	−	−	−	−
	Caffeic acid	−	−	−	−	−	−	−	−	−
	Cryptochlorogenic acid	−	−	−	−	+	+	−	−	+
	4-Coumaric acid	−	−	−	−	−	−	−	−	−
	3,5-di-O-Caffeoyl quinic acid	−	−	+	−	−	−	−	−	−
	1,5-di-O-Caffeoyl quinic acid	−	−	+	−	−	−	−	−	−
	3,4-di-O-Caffeoyl quinic acid	−	−	−	−	−	−	−	−	−
	Rosmarinic acid	−	+	−	−	−	+	+	−	−
flavonols	Myricetin-3-O-rutinoside	−	−	−	−	+	+	−	−	+
	Myricetin-3-O-glucoside	−	−	−	−	+	+	−	−	+
	Quercetin-3-O-rutinoside	−	−	−	−	−	−	−	−	−
	Quercetin-3-O-glucoside	−	−	−	−	−	−	−	−	−
	Quercetin-3,4′-di-O-glucoside	−	−	−	−	−	−	−	−	−
	Kaempferol-3-O-rutinoside	−	−	−	−	−	−	−	−	−
anthocyanins	Delphinidin-3-O-glucoside	−	−	−	−	+	+	−	−	+
	Dephinidin-3-O-rutinoside	−	−	−	−	+	+	−	−	+
	Cyanidin-3-O-glucoside	−	−	−	−	+	+	−	−	+
	Cyanidin-3-O-rutinoside	−	−	−	−	+	+	−	−	+

**Table 3:** Changes in color, pH, and gel strength of myofibrillar protein samples supplemented with plant-based additives.

	Color			pH	Gel Strength [N]
	L	a	b		
Control: Myofibrillar protein without additives	76.36 ^c^ ± 1.82	−0.92 ^ba^ ± 1.20	18.59 ^dc^ ± 2.06	6.17 ^b^ ± 0.01	0.87 ^c^ ± 0.28
Myofibrillar protein + black currant pomace	73.33 ^bc^ ± 2.49	2.59 ^c^ ± 1.28	15.99 ^bc^ ± 3.60	6.25 ^c^ ± 0.01	0.27 ^a^ ± 0.29
Myofibrillar protein + black currant juice	67.73 ^ba^ ± 4.50	0.23 ^bca^ ± 0.41	12.00 ^a^ ± 1.01	6.23 ^c^ ± 0.02	0.42 ^ab^ ± 0.15
Myofibrillar protein + *M. officinalis* extract	70.52 ^b^ ± 0.26	−1.75 ^a^ ± 0.05	14.03 ^ab^ ± 1.16	6.13 ^a^ ± 0.01	0.36 ^ab^ ± 0.29
Myofibrillar protein + *M. officinalis* extract + black currant pomace	68.18 ^ba^ ± 2.81	1.23 ^bc^ ± 1.50	20.69 ^d^ ± 0.67	6.13 ^a^ ± 0.01	0.20 ^a^ ± 0.14
Myofibrillar protein + *M. officinalis extract* + black currant juice	68.53 ^ba^ ± 4.19	0.56 ^bca^ ± 0.84	20.15 ^d^ ± 1.04	6.13 ^a^ ± 0.00	0.28 ^a^ ± 0.12
Myofibrillar protein + *C. asiatica* extract	69.60 ^ba^ ± 3.94	0.16 ^bca^ ± 0.85	21.45 ^d^ ± 0.74	6.14 ^a^ ± 0.01	0.55 ^b^ ± 0.21
Myofibrillar protein + *C. asiatica* extract + black currant pomace	64.28 ^a^ ± 3.25	1.56 ^bc^ ± 3.39	21.73 ^d^ ± 2.33	6.37 ^d^ ± 0.02	0.33 ^ab^ ± 0.25
Myofibrillar protein + *C. asiatica* extract + black currant juice	71.31 ^bc^ ± 1.98	0.05 ^bca^ ± 1.37	28.20 ^e^ ± 3.99	6.26 ^c^ ± 0.04	0.21 ^a^ ± 0.17
*p*	0.008	0.007	0.000	0.000	0.000
SEM	0.811	0.339	0.955	0.015	0.036

Values in the same column with different uppercase letters (a, b, c, etc.) indicate statistically significant differences between means (*p* < 0.05), determined by post hoc multiple comparison tests. Identical letters denote groups that do not differ significantly. The *p*-value (*p*) represents the significance level of the statistical test, where *p* < 0.05 indicates significant differences among samples. The standard error of the mean (SEM) reflects the precision of the mean estimate, with lower values indicating greater reliability of the measurement.

**Table 4:** Sensory (color, flavor, consistency, structure, and taste) changes across the various myofibrillar protein samples supplemented with plant-based additives.

	Color	Flavor	Consistency	Structure	Taste
Control: Myofibrillar protein without additives	4.50 ^bc^ ± 0.84	2.83 ^a^ ± 1.33	4.43 ^d^ ± 0.79	4.43 ^d^ ± 0.79	4.00 ^d^ ± 0.58
Myofibrillar protein + black currant pomace	3.50 ^ba^ ± 0.84	3.17 ^a^ ± 1.33	3.43 ^abcd^ ± 0.79	3.57 ^abcd^ ± 0.79	3.50 ^dcab^ ± 0.76
Myofibrillar protein + black currant juice	3.07 ^a^ ± 0.84	3.20 ^a^ ± 1.10	2.67 ^a^ ± 1.03	2.83 ^ab^ ± 0.98	2.83 ^ab^ ± 0.52
Myofibrillar protein + *M. officinalis* extract	3.17 ^a^ ± 0.98	2.83 ^a^ ± 0.98	3.00 ^abc^ ± 1.22	2.80 ^a^ ± 0.84	2.75 ^a^ ± 0.76
Myofibrillar protein + *M. officinalis* extract + black currant pomace	4.50 ^bc^ ± 0.84	3.25 ^a^ ± 1.41	4.00 ^bcd^ ± 1.00	4.14 ^cd^ ± 0.69	3.86 ^d^ ± 0.85
Myofibrillar protein + *M. officinalis* extract + black currant juice	3.80 ^bca^ ± 0.45	3.60 ^a^ ± 1.14	3.60 ^abcd^ ± 0.89	3.60 ^abcd^ ± 0.89	3.70 ^dcb^ ± 0.57
Myofibrillar protein + *C. asiatica* extract	4.17 ^bc^ ± 0.41	3.33 ^a^ ± 0.82	3.50 ^abcd^ ± 1.05	3.17 ^abc^ ± 0.75	3.75 ^dc^ ± 0.42
Myofibrillar protein + *C. asiatica* extract + black currant juice	4.57 ^c^ ± 0.79	3.71 ^a^ ± 0.76	4.14 ^cd^ ± 0.69	3.88 ^bcd^ ± 0.99	3.81 ^dc^ ± 0.92
Myofibrillar protein + *C. asiatica* extract + black currant pomace	3.57 ^bca^ ± 0.79	3.14 ^a^ ± 1.07	2.83 ^ab^ ± 0.75	2.71 ^a^ ± 0.76	2.94 ^cab^ ± 0.82
*p*	0.002	0.892	0.013	0.002	0.008
SEM	0.123	0.145	0.137	0.128	0.106

Values in the same column with different uppercase letters (a, b, c, etc.) indicate statistically significant differences between means (*p* < 0.05), determined by post hoc multiple comparison tests. Identical letters denote groups that do not differ significantly. The *p*-value (*p*) represents the significance level of the statistical test, where *p* < 0.05 indicates significant differences among samples. The standard error of the mean (SEM) reflects the precision of the mean estimate, with lower values indicating greater reliability of the measurement.

## Data Availability

The original contributions presented in the study are included in the article, further inquiries can be directed to the corresponding author.
